# Celebrating Two Centuries of Research in Selenium Chemistry: State of the Art and New Prospective

**DOI:** 10.3390/molecules22122124

**Published:** 2017-12-02

**Authors:** Claudio Santi, Luana Bagnoli

**Affiliations:** Group of Catalysis and Organic Green Chemistry, Department of Pharmaceutical Sciences University of Perugia, Via del Liceo 1, 06134 Perugia, Italy; luana.bagnoli@unipg.it

**Keywords:** selenium, catalysis, green chemistry, antioxidants, antimicrobial, functional nutrients

## Abstract

In 2017, the 200th anniversary of the discovery of selenium was celebrated. In 1817, the Swedish chemists, Berzelius and Gahn, on roasting 200 kg of sulfur from a pyrite from the Falun mine, obtained about 3 g of a precipitate that they first wrongly identified as tellurium. Berzelius doubted this result and repeated the analysis some months later realizing that a new element was in his hands and he named this element Selenium (Greek: Selene, moon) in consideration of its resemblance to Tellurium (Latin: Tellus, earth). Several events were organized in the year for this special celebration and this Special Issue would like to be an additional contribution to the success of a research that, especially during the last decades, rapidly grew in different fields: synthesis, medicinal chemistry, biology, material, and environment. These studies are strongly characterized by multi- and interdisciplinary connections, and, for this reason, we collected here contributions coming from different areas and disciplines, not exclusively synthetic organic chemistry.

For a long time, the main biological activity of selenium derivatives was supposed to be connected to their toxicity and some exotic theories correlated to historical events of selenium poisoning. Even if, nowadays, several studies have confirmed that some disorders in animals and humans are a consequence of selenium bioaccumulation, it is also clear that selenium is fundamental for life. Nowadays, it is known that selenium is an essential micronutrient and that its presence in 25 selenoproteins have a crucial role in the redox equilibrium of living systems. For this reason, the biochemistry and potential biological effects of organoselenium compounds are becoming an attractive and even more promising area of investigations [1]. From a synthetic point of view, the versatility of organoselenium reagents offers the possibility to perform highly selective reactions normally using mild conditions. Furthermore, in the recent past, the possibility to set up efficient catalytic protocols using non-conventional and ecofriendly medium opened the way for the application of selenium derivatives in green chemistry [2].

In 2017, several events were dedicated to the celebration of this important anniversary: Xth International Mini-Symposium “Selenium containing compounds on the borderline of chemistry, biology, and medicine” Lodz University (May), the joint meeting between the 11th International Symposium on Selenium in Biology and Medicine and the 5th International Conference on Selenium in the Environment and Human Health (Se2017) in Stockholm (August); the Symposium on Selenium Chemistry & Biology (SSCB-17) in Mumbai (November); and, in September, the 6th Workshop of the international Network Selenium Sulfur and Redox Catalysis (WSeS-6) in Wroclaw (Poland). On this last occasion, awards (supported by *Molecules*) to young researchers/students dedicated to the memory of two eminent scientist in the field of organoselenium chemistry were assigned: Ms Francesca Pensa received the “*Nicola Petragnani Award*” for her work in the Synthesis and characterization of a novel enantiomerically pure diselenide and its application in asymmetric synthesis; and Mr Bonifacio Monti received the “*Marcello Tiecco Award*” for his work on the reagents for the ring opening of epoxide under non-conventional conditions. 

This Special Issue aims to be an additional contribution to the celebrations, collecting 16 papers (three review articles and 13 original papers) from outstanding research groups covering different aspects of the research in the field: synthesis, catalysis, green chemistry, new biologically active compounds, biological evaluation of selenium derivatives, nutrition and environmental speciation. Woolins’ group reported here the synthesis and characterization of a series of new 4-substituted-1,3-selenazol-2-amines. The synthesis was carried out by two-component cyclization of the selenoureas and α-haloketones and the selenoureas were obtained from the reaction of Woollins’ reagent with cyanamides, followed by hydrolysis [3]. Other contributions pay attention to the ecofriendly aspect of synthesis, such as the paper from Braga et al. [4] that described an efficient copper-catalyzed synthesis of unsymmetrical disubstituted chalcogenides using ligand- and solvent-free conditions; our manuscript, in which the use of novel zinc selenates was proposed for the preparation in “on-water” conditions of selenol esters [5]; and the efforts of Lenardao’s and Perin’s groups who suggested the use of glycerol as ecofriendly raw material in the synthesis of organoselanyl and organotellanyl alkynes [6] or the green hydroselenation of aryl alkynes to afford divinyl selenides as precursor in the synthesis of resveratrol [7]. The preparation of novel organoselenium compound having biological activity is here approached by Scianowski’s group with the synthesis of new cytotoxic Ebselen-like derivatives [8] as well as by Giurg and coworkers who prepared different derivatives of bis[(2-chlorocarbonyl)phenyl] diselenide demonstrating their antibacterial, antifungal and antiviral properties [9].

Interestingly, Iwaoka’s group investigated the GPx-like mechanism of some water soluble amino substituted selenides, observing that, in methanol, the catalytic cycle is shifted with respect to water. In our opinion, this confirms the growing perplexity on the methods used since now for the comparison and, sometimes, the evaluation of the GPx-like activity of selenium compounds [10].

Yuan et al. reported the anti-mutagenic effects of selenium-enriched polysaccharides from *Pyracantha fortuneana* suggesting its use as an alternative strategy for cancer therapy by targeting CYP1A family [11], while Wang’s group demonstrated that high levels of Se could protect against reproductive system damage in male mice caused by zearalenone through a double mechanism: improving antioxidant ability and reducing the apoptosis of the reproductive cells [12]. Interestingly, it was also proven that selenium nanoparticles can attenuate diabetes-induced oxidative damage, in particular in testicular tissue [13]. An interesting study on the determination of selenium and selenomethionine content in the biomass of feed yeast *Candida utilis* ATCC 9950 is here reported by Kieliszek et al., demonstrating that the proposed protocol represents an interesting way to obtain selenium biocomplexes to be used as dietary supplements [14]. Crans et al. from Colorado State University optimized the conditions for speciation analysis of the creek waters in three of the main tributaries in Colorado, USA. The possibility of controlling not only the amount of selenium but also its chemical nature is an important aspect in the process of environmental protection allowing for additional insights into the processes in a hydrological system [15].

Finally, the review articles cover a broad area of scientific interests: *synthesis*, with a collection of recent methods for performing electrophilic selenium catalyzed functionalization of alkenes in the presence of N-F reagents as oxidants proposed by Zhao et al. with a critical analysis of the still unexplored aspects of this kind of chemistry [16]; *nutrition*, with an overview of the importance of selenium in food and the need to supplement carefully and cautiously proposed by Marek Kieliszek [17]; and *agriculture*, with a description of the role of selenium enrichment of horticultural crops as a strategy to preserve a longer shelf-life and longer-lasting properties reported by Pezzarosa et al. [18].

In conclusion, we were honored to act as Guest Editors of this nice and inspiring Special Issue; we thank all of the outstanding authors for their efforts and their collaborative participation; all of the referees who ensured the good quality of the publications. Furthermore, we hope this Special Issue can attract the interest of a broad panel of readers in terms of disciplines and scientific interests because we strongly convinced that, even after 200 years, the research around selenium chemistry, biology and medicine will continue to be exciting and productive for a long period. 
